# Microhabitat use, population densities, and size distributions of sulfur cave-dwelling *Poecilia mexicana*

**DOI:** 10.7717/peerj.490

**Published:** 2014-07-15

**Authors:** Jonas Jourdan, David Bierbach, Rüdiger Riesch, Angela Schießl, Adriana Wigh, Lenin Arias-Rodriguez, Jeane Rimber Indy, Sebastian Klaus, Claudia Zimmer, Martin Plath

**Affiliations:** 1 Evolutionary Ecology Group, Goethe University of Frankfurt, Frankfurt am Main, Germany; 2 Biodiversity and Climate Research Centre (BiK^F^), Frankfurt am Main, Germany; 3 Leibniz-Institute of Freshwater Ecology and Inland Fisheries, Berlin, Germany; 4 Department of Animal and Plant Sciences, University of Sheffield, Sheffield, UK; 5 División Académica de Ciencias Biológicas, Universidad Juárez Autónoma de Tabasco (UJAT), Villahermosa, Tabasco, México

**Keywords:** Cave fish, Extremophile teleosts, Fisheries, Rotenone, Overcompensation

## Abstract

The Cueva del Azufre in Tabasco, Mexico, is a nutrient-rich cave and its inhabitants need to cope with high levels of dissolved hydrogen sulfide and extreme hypoxia. One of the successful colonizers of this cave is the poeciliid fish *Poecilia mexicana*, which has received considerable attention as a model organism to examine evolutionary adaptations to extreme environmental conditions. Nonetheless, basic ecological data on the endemic cave molly population are still missing; here we aim to provide data on population densities, size class compositions and use of different microhabitats. We found high overall densities in the cave and highest densities at the middle part of the cave with more than 200 individuals per square meter. These sites have lower H_2_S concentrations compared to the inner parts where most large sulfide sources are located, but they are annually exposed to a religious harvesting ceremony of local Zoque people called La Pesca. We found a marked shift in size/age compositions towards an overabundance of smaller, juvenile fish at those sites. We discuss these findings in relation to several environmental gradients within the cave (i.e., differences in toxicity and lighting conditions), but we also tentatively argue that the annual fish harvest during a religious ceremony (La Pesca) locally diminishes competition (and possibly, cannibalism by large adults), which is followed by a phase of overcompensation of fish densities.

## Introduction

Cave fishes are emerging as model systems to study regressive evolutionary processes like the reduction of eyes and pigmentation that typically accompany the colonization of caves by previously surface-dwelling species (Romero & Green, 2005; Jeffery, 2009). For example, the characid *Astyanax mexicanus* is a model organism for EvoDevo studies of cave evolution (Wilkens, 1988; Jeffery, 2001; Jeffery, 2009). The cave form of a Mexican live-bearing fish, the so-called cave molly (*Poecilia mexicana*; Gordon & Rosen, 1962) has adapted to the vastly divergent ecological conditions inside a South Mexican sulfide cave, the Cueva del Azufre (also referred to as Cueva Villa Luz or Cueva de las Sardinas; Parzefall, 1993; Parzefall, 2001) Cave environments are usually energy limited compared to photosynthetically based epigean habitats (Hüppop, 2005) and fish densities reported for several different cave systems are low, with often less than one individual per m^2^ (Trajano, 2001). In contrast, the Cueva del Azufre is a sulfidic, nutrient-rich habitat due to chemoautotrophic primary production through sulfide oxidizing bacteria that utilize the abundant hydrogen sulfide in the cave (Hose & Pisarowicz, 1999; Colaço, Dehairs & Desbruyeres, 2002; Summers Engel, 2005). Hydrogen sulfide is acutely toxic to most metazoans and leads to extreme hypoxia in the water (Evans, 1967; Bagarinao, 1992). Beside the Cueva del Azufre, few other sulfurous chemoautotrophic cave-ecosystem are described, such as Movile in Romania (Sarbu, Kane & Kinkle, 1996), Frasassi in Italy (Flot, Wörheide & Dattagupta, 2010) and Ayyalon in Israel (Por, 2007). All of these caves are inhabited by invertebrates—many of them endemic to the caves—that exploit this unusual food web. The Cueva del Azufre is the only known chemoautotrophic cave ecosystem which is inhabited by a vertebrate species (Plath & Tobler, 2010). However, due to its toxicity, hydrogen sulfide requires energetically costly behavioral (i.e., actively avoiding microhabitats with high levels of toxicity) and physiological adaptations (various forms of detoxification) by animals exposed to it (Tobler et al., 2009; Riesch, Plath & Schlupp, 2010). As a result of the simultaneous action of two strong selective forces (permanent darkness and hydrogen sulfide), locally adapted *P. mexicana* populations in the Cueva del Azufre system have received considerable scientific interest. The cave molly differs from its surface-dwelling ancestors in a distinct set of morphological, physiological, behavioral, and life-history traits; e.g., cave mollies have reduced eye size and reduced pigmentation, and females have a reduced fecundity combined with an increase in individual offspring size (Parzefall, 2001; Tobler et al., 2008a; Riesch, Plath & Schlupp, 2010; Tobler et al., 2011b). Although the cave molly has been established as a model to examine evolutionary adaptations to extreme environmental conditions, population densities have not yet been quantified in the Cueva del Azufre system, which makes interpretation of some of the ecological and evolutionary data difficult with regards to how they influence long-term stability of the systems and population dynamics.

The Cueva del Azufre drains into the El Azufre, a sulfidic surface creek, which eventually joins the Río Oxolotán. The Cueva del Azufre and El Azufre differ dramatically in the composition of fish communities compared to adjacent non-sulfidic surface habitats. *Poecilia mexicana* occurs as the single dominant species in both systems. Only one further fish species, the predatory cichlid *‘Cichlasoma’ salvini* occurs in the upper parts of the El Azufre, but only in small numbers. In downstream areas of the El Azufre where H_2_S in not measurable, *Heterandria bimaculata* and *Xiphophorus hellerii* (Poeciliidae), *Astyanax aeneus* (Characidae) as well as ‘*Cichlasoma’ salvini* and *Thorichtys helleri* (Cichlidae) occur (Plath & Tobler, 2010). In surrounding non-sulfidic surface habitats, diverse fish communities can be found, often dominated by cichlid and poeciliid species (Tobler et al., 2006; Plath & Tobler, 2010). In the Clear Creek, a small stream that is directly connected to El Azufre, *H. bimaculata* occurs at a high abundance together with small numbers of *X. hellerii* and *P. mexicana.* A reduced species diversity and dominance of a few specialists have been documented from other caves (Trajano, 2001) and other sulfidic habitats (Tobler et al., 2008c).

**Figure 1:**
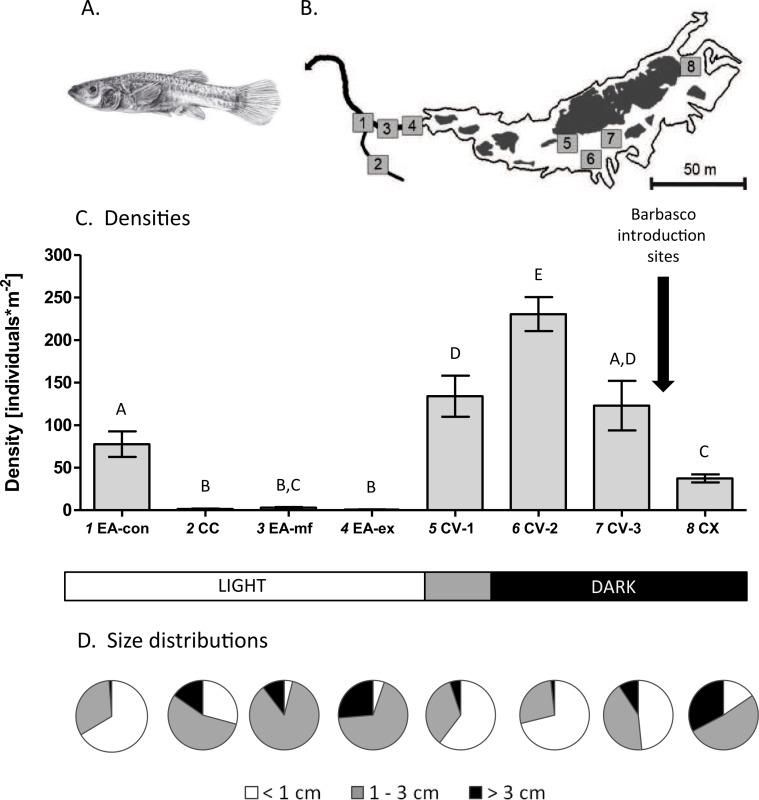
Study system and population densities. (A) Drawing of a female cave molly. (B) Map of the study area showing the different sampling sites (*numbers*) where white areas represent water within the cave (Cueva del Azufre) and dark areas indicate dry land and bedrock. *1* EA-con, *2* CC, *3* EA-mf, *4* EA-ex, *5* CV-1, *6* CV-2, *7* CV-3, *8* CX. With the exception of sampling site CV-1 all sampling sites inside the Cueva del Azufre are completely dark. Barbasco is released annually between chamber V (CV) and chamber X (CX). Three sampling sites inside chamber V were defined (CV-1 to CV-3). Downstream of the exit of the Cueva del Azufre (EA-ex), a rather homogeneous mudflat (EA-mf) was sampled. Further sampling sites were a small non-sulfidic creek (Clear Creek; CC) and its confluence with El Azufre (EA-con). (C) Mean (±SE) densities of mollies at each sampling site. Letters above the error bars signify statistically different groups (Fisher’s LSD tests). (D) Size class compositions of mollies at the different sampling sites.

Little is known about anthropogenic disturbances on the population ecology of *P. mexicana* inhabiting the Cueva del Azufre. Today, the system is increasingly influenced by a growing number of visitors which reach their peak during a traditional annual ceremony of the local indigenous Zoque people named ‘La Pesca’. The Cueva del Azufre is sacred to the Zoque people, and once a year, on the first Sunday of Easter week, the Zoque enter the cave and introduce rotenone- and deguelin-containing barbasco roots (*Lonchocarpus* sp., Fabaceae) into the water. Rotenone is an inhibitor of the mitochondrial complex-I of the respiratory chain, causing reduced cellular respiration (Singer & Ramsay, 1994). Barbasco is introduced into the water in the middle portion of the cave, therefore only downstream cave chambers are affected (Fig. 1). Capture of poisoned cave fish is facilitated by the anesthetic effect of barbasco, as narcotized fish are flushed out of the cave, where they are harvested using wooden baskets, and afterwards cooked and eaten as part of a religious ceremony honoring the Rain Gods (Tobler et al., 2011a). The yield of the annual harvest is considered to be indicative of the quality of the subsequent crop harvest (Hose & Pisarowicz, 1999; Tobler et al., 2011a). Annual harvests amount to several thousand individuals, and the ceremony is likely to have taken place for centuries (Hose & Pisarowicz, 1999), so it is likely to act as a strong selective force on *P. mexicana* populations annually exposed to it.

In the present study, we provide first data of local densities within different chambers of the Cueva del Azufre and adjacent El Azufre and discuss our findings with regard to environmental conditions and annual harvesting of cave mollies. We used a non-invasive technique to repeatedly assess fish densities and size-distribution patterns (as a proxy for age) inside the Cueva del Azufre (up- and downstream of the barbasco-release site) and in the sulfidic creek leaving the cave (El Azufre). Moreover, given the high structural heterogeneity of the water course inside the Cueva del Azufre with respect to water depth and flow velocity (Hose & Pisarowicz, 1999), and because Croft, Botham & Krause (2004) reported on size-specific preferences regarding water depth in another poeciliid, the Trinidadian guppy (*P. reticulata*), we combined our assessment of fish densities with an investigation of microhabitat use by different size classes of cave mollies.

## Material and Methods

### Study system

Locally adapted subterranean populations of *P. mexicana* (Fig. 1A) can be found in at least two different limestone caves in the vicinity of the southern Mexican city of Tapijulapa (state of Tabasco, México): the Cueva del Azufre (Gordon & Rosen, 1962) and the much smaller, non-sulfidic Cueva Luna Azufre (Tobler et al., 2008b). The sulfidic Cueva del Azufre is about 500–600 m deep and divided into 13 different cave chambers (I-XIII), with the innermost chamber being XIII (Gordon & Rosen, 1962). Several springs discharge water with high concentrations of hydrogen sulfide (H_2_S) into the creek draining the cave (Tobler et al., 2006). The cave creek forms a highly heterogeneous mosaic of shallow pools and backwaters that are partially divided by swift flowing riffle passages (Gordon & Rosen, 1962; Hose & Pisarowicz, 1999). While the front cave chambers receive some dim light through cracks in the ceiling, the inner parts of the cave are lightless. Consequently, (sub-)populations experience divergent selection regimes regarding light exposure, with populations from the innermost chambers living under perpetually dark conditions, whereas those from front chambers are exposed to dim sunlight through a number of cracks in the cave ceiling, so-called sky lights (Fontanier & Tobler, 2009).

Upon leaving the underground, the sulfidic creek draining the Cueva del Azufre is called ‘El Azufre’. After meandering for approximately 1.5 km, it eventually drains into the Río Oxolotán, which is part of the Río Grijalva drainage system. Despite the gradual oxidation of H_2_S to sulfate and elemental sulfur with increasing distance from the sulfide sources, which increases the water turbidity, and despite the influx of some smaller clear water affluents, El Azufre still has a remarkably high H_2_S concentration of up to ∼40 µMol (Tobler et al., 2006; Schlupp et al., 2013).

### Study sites and data collection

**Figure 2:**
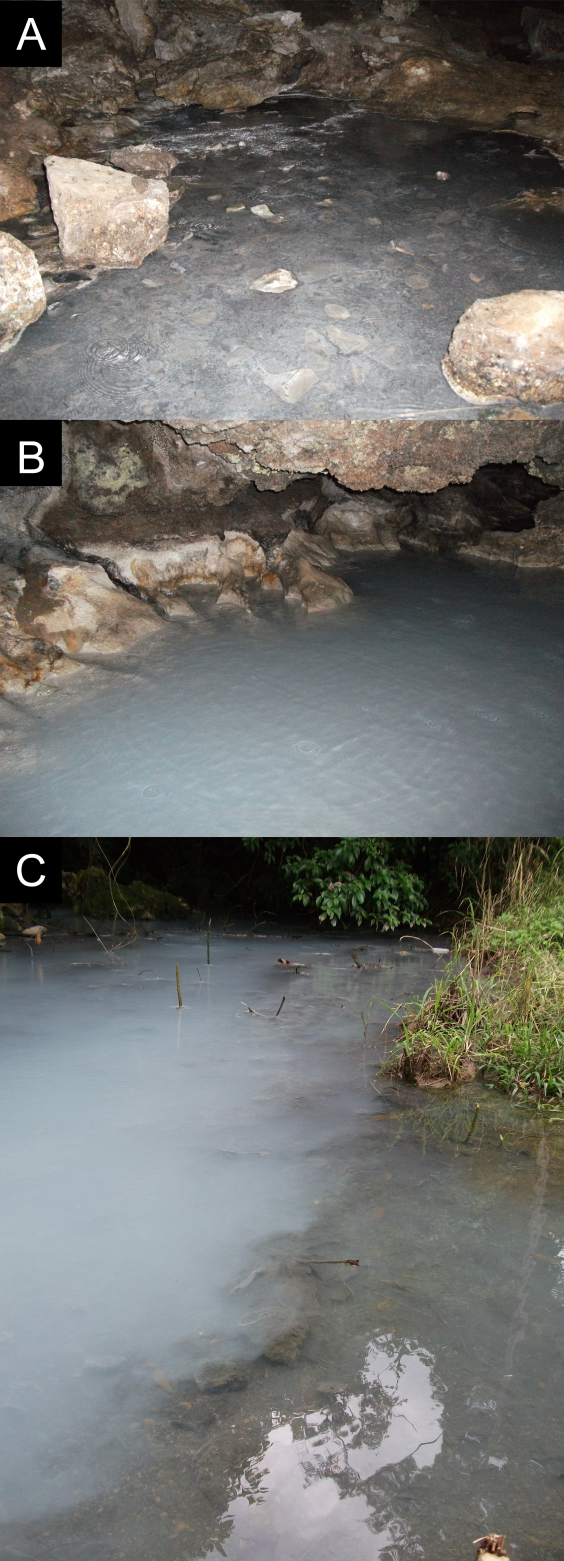
Pictures of study sites. (A) Cueva del Azufre chamber V (*6* CV-2) and (B) site *7* CV-3. (C) El Azufre confluence with Clear Creek (*1* EA-con).

We compared the abundance and distribution of different size classes of *P. mexicana* among different sampling sites along a transect starting at chamber X in the Cueva del Azufre, and following the water flow outside the cave to the confluence of El Azufre with the first freshwater influx from the Clear Creek. This transect, therefore, covered sample sites located upstream of the release point of barbasco during La Pesca (sample point in chamber X) and sites directly downstream of the release point of barbasco that are strongly affected by the annual ceremony (three sites in chamber V; CV-1, CV-2, and CV-3; Figs. 2A and 2B). Surface sites of El Azufre are also annually exposed to barbasco due to downstream effects (EA-ex, EA-mf), even though concentrations are probably considerably lower than inside the cave (Table 1). Clear creek (CC) and its confluence with EA (EA-con; Fig. 2C), on the other hand, are not influenced by barbasco.

**Table 1:** Sampling sites, their abbreviation code as used throughout the article, numbers of quadrants examined, and details regarding barbasco release.

Site code	Site	Number of quadrants	Affected by deposition of rotenone?	Approximate distance to upstream rotenone release site [m]
*1* EA-con	El Azufre, confluence with clear Creek	25	No (only partly)	150
*2* CC	Clear Creek	28	No	—
*3* EA-mf	El Azufre, mudflat	25	Yes	120
*4* EA-ex	El Azufre, exit of the Cueva del Azufre	25	Yes	110
*5* CV-1	Cueva del Azufre, Chamber V, site 1	25	Yes	0
*6* CV-2	Cueva del Azufre, Chamber V, site 2	15	Yes	0
*7* CV-3	Cueva del Azufre, Chamber V, site 3	8	Yes	0
*8* CX	Cueva del Azufre, Chamber X	25	No	—

**Figure 3:**
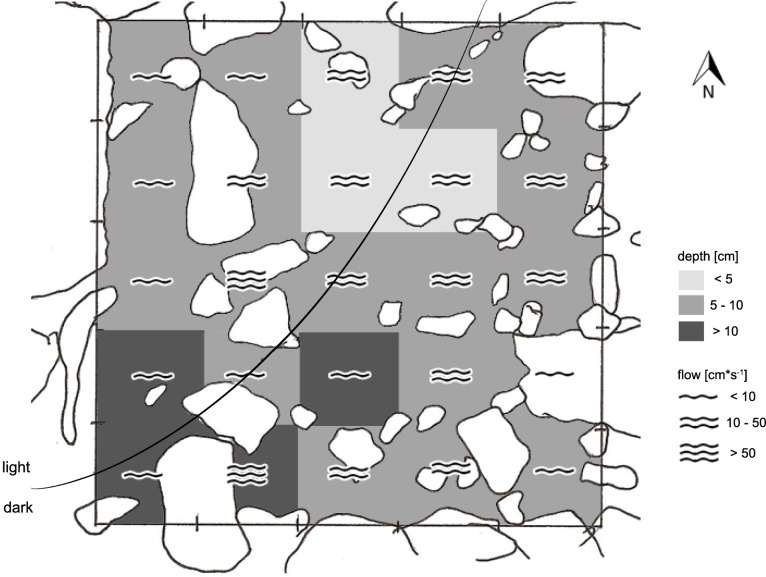
Exemplary sketch of site *5* CV-1. Showing the high degree of heterogeneity in flow regimes, water depth, substrate types, and (in this case) light regime.

Field work was conducted in January 2010, i.e., about 9 month after the latest La Pesca ceremony in 2009 (L Arias-Rodriguez, pers. obs., 2009). At each of the eight sample sites, we defined sampling grids consisting of 50 × 50 cm quadrants with wooden sticks fixed in the ground (or stones where a grid angle fell on the shore). The number of quadrants was mostly 25 per sampling grid (i.e., 5 × 5 quadrants). In the narrow non-sulfidic surface creek (CC), however, the arrangement of quadrants was more longitudinal (4 × 7 = 28 quadrants), and in chamber V, where a particularly high degree of structural heterogeneity precluded defining larger grids, one sampling site of 5 × 5 quadrants and two smaller ones (15 and 8 quadrants, respectively) were defined (Table 1). The grids reflected the natural variation in water depth, flow velocity, and substrate types, thus covering the range of different microhabitats inhabited by mollies (an example is shown in Fig. 3).

Daily measurements took place between 11:00 a.m. and 4:30 p.m. Each site was visited at least 5 times (mean ± SD = 6.25 ± 1.16) on consecutive days. During the counts, we slowly approached a site while trying to avoid any movements that would cause the resident fish to flee, and we counted juveniles (<10 mm standard length (SL)), sub-adults (10–30 mm) and adults (>30 mm) in each quadrant. The observer was standing motionless at least 1.5 m downstream from the respective quadrant. Sizes were estimated qualitatively, aided by a prior training session that used wooden sticks of known size as a reference. Our definition of adults roughly followed Riesch, Plath & Schlupp (2010), who determined the mean (±SD) standard length of reproducing females to be 31.44 ± 4.40 mm (El Azufre) and 36.97 ± 4.59 mm (Cueva del Azufre, chambers V and X).

Habitat parameters were assessed after the last fish count. For each quadrant, we determined water depth using a wooden ruler stuck vertically into the water at five random locations and calculating the mean from those five measurements. Flow velocity was measured on the water surface by scoring the time a small wooden stick of about 3 cm length took to float through the whole length of a quadrant (measurement was repeated five times per quadrant and averaged across the five observations per quadrant). Mean surface flow velocity was then expressed as cm*s^−1^. Research followed the authorizations from CONAPESCA-DGOPA.09004.041111.3088 and Tacotalpa, Tabasco municipality.

### Statistical analysis

Our first question was whether population densities differed among sampling sites. We used data for the different quadrants per site (averaged from the repeated measurements) and expressed density as total numbers of individuals per square meter. Density estimates per quadrant were used as the dependent variable in a univariate general linear model (GLM) with ‘sampling site’ as a fixed factor. We initially entered ‘mean water depth’ (*F*_1,162_ = 0.12, *P* = 0.98) and ‘mean flow rate’ (*F*_1,162_ = 0.22, *P* = 0.64) as covariates, but removed them from the final analysis since neither had a significant effect (also none of the interaction terms were significant). We used Fisher’s LSD tests for pairwise *post hoc* comparisons among sites.

A further question was whether size-class compositions differed among sample sites and whether distribution patterns would be stable among repeated sampling days. We used the Bray-Curtis index (Bray & Curtis, 1957) to estimate pairwise similarities among each sampling point (calculated with the R-package ‘ecodist 1.2.7’; Goslee and Urban, 2007; R Development Core Team, 2008), and used these for non-metric multidimensional scaling (‘NMDS PROXSCAL’ function in SPSS 21). To detect clusters, we used the ‘two step cluster analysis’ function based on Euclidian distances and the Bayesian information criterion. For visualization of size class compositions per site, we averaged repeated measurements of different size classes and used a mean value for each quadrant per site. By using these means we calculated the total average size class distribution per site.

Our first analysis detected pronounced variation in population densities and size distributions (see results) and thus, we decided to analyze potential effects of water depth and flow velocity (i.e., microhabitat choice) in a site-wise fashion. We focused on sites inside the Cueva del Azufre (CV-1, CV-3 and CX) where (*a*) fish densities were sufficiently high and (*b*) sufficient variability of those environmental variables was found to allow for a meaningful analysis. All other sites were excluded from this analysis. For each site, fish density per quadrant was entered as the dependent variable in repeated measures (rm) GLMs with ‘size class’ (three levels) as the repeated measurement. We grouped water depth (<5 cm, 5–10 cm, >10 cm) and flow velocity (<10 cm*s^−1^, 10–50 cm*s^−1^, >50 cm*s^−1^) into three classes each and used these habitat parameters as fixed factors. However, neither the main factor ‘flow velocity’ nor any interaction term involving ‘flow velocity’ had a significant effect in any of the three site-specific models (CV-1: *F*_4,38_ = 1.27, *P* = 0.30; CV-3: *F*_2,4_ = 3.28, *P* = 0.14; CX: *F*_2,20_ = 0.44, *P* = 0.65), and so we subsequently removed this term from all models.

## Results

### Local population densities

When comparing mean densities per quadrant across sites we detected a significant difference among sampling sites (GLM; *F*_7,164_ = 32.49, *P* < 0.001; Fig. 1B). *Post hoc* pairwise LSD tests found most pairwise comparisons to be statistically significant; qualitatively, densities increased from surface sites (mean ± SE across sites = 21.0 ± 5.0 individuals*m^−2^) towards the cave (119.5 ± 12.7 individuals*m^−2^). Also, sites downstream of the barbasco release-site (chamber V; 162.3 ± 16.1 individuals*m^−2^) had considerably higher densities than the site in chamber X that lies upstream of the release-site (37.4 ± 4.8 individuals*m^−2^).

### Differences in size-class compositions

**Figure 4:**
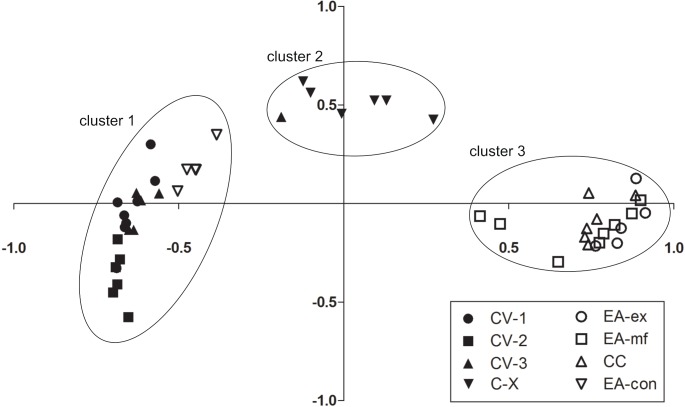
Differences in size-class compositions of *Poecilia mexicana* in the Cueva del Azufre system. Non-metric Multi-Dimensional Scaling (NMDS) plots based on Bray-Curtis similarities for each sampling site and day.

The NMDS based on Bray-Curtis similarities found data from repeated sampling days to cluster together, suggesting that the observed size-class compositions were stable over the period of this study (Fig. 4). There were three distinct clusters, and in only one case was a single sampling day of a given sampling site assigned to the ‘wrong’ cluster. The first cluster comprised the three sample sites in cave chamber V and EA-con. Samples had high overall densities and were composed mostly of small individuals. Cluster two comprised the rearmost cave site CX. Samples in this cluster were characterized by intermediate densities but a particularly high proportion of large individuals. Cluster three comprised all surface sites except EA-con and was characterized by overall low densities and mostly intermediate-sized fish (Fig. 4).

### Microhabitat use of different size classes

**Figure 5:**
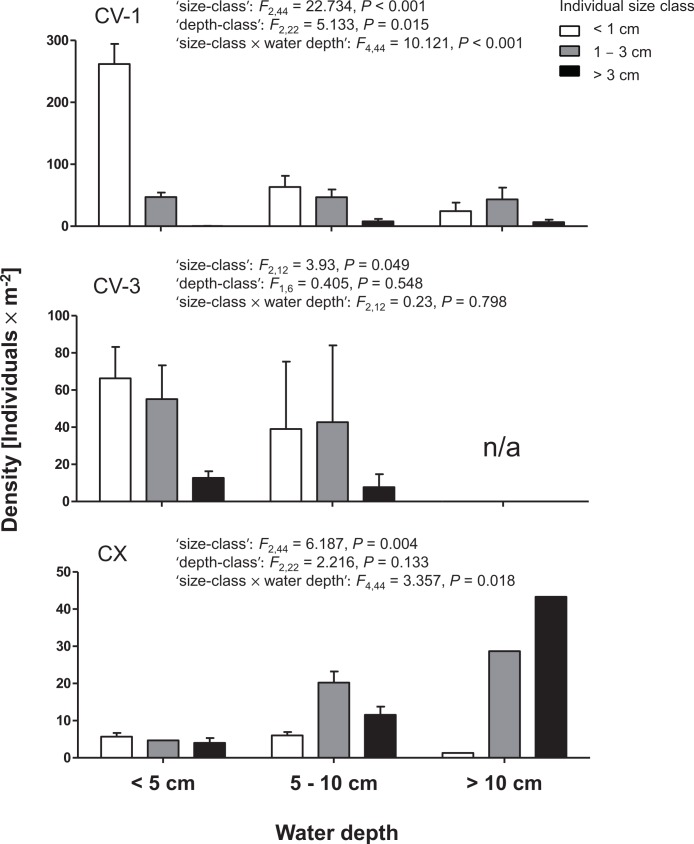
Population densities of cave mollies in the Cueva del Azufre. Mean (± SE) densities of mollies, categorized in three size classes (<1 cm, *white*, 1–3 cm, *gray*, and >3 cm, *black*) in three water depths (<5 cm, 5–10 cm, and >10 cm). Results of rmGLMs are inserted. Note the different y-axis scales. Error bars are given if more than one sampling grid of a given depth class was present within the sampling site.

In the rmGLMs treating the different size-classes as the repeated measurement, the interaction term ‘size-class × water-depth’ had a significant effect for two of the three sampling sites included in this analysis—notably, those sites with the most variation in water depth (Fig. 5). This result is indicative of differences in microhabitat use among different size classes of cave mollies: generally, larger fish were found in deeper areas, whereas smaller fish resided in shallow parts. A significant main effect of the repeated measurement (‘size class’) in all three analyses confirms the overabundance of small-sized fish in cave chamber V, and of large-bodied fish in chamber X (Fig. 5).

## Discussion

We provide detailed information on population densities of cave-adapted *P. mexicana* in the Cueva del Azufre. Repeated measurement in different cave chambers uncovered very stable patterns of high densities, confirming qualitative estimates provided by Parzefall (1993). Density estimates of *P. mexicana* in the cave were extraordinarily high and exceed those of other cave fishes, which are usually low, with often less than one individual per m^2^ (Trajano, 2001). Furthermore, densities were higher inside the cave compared to adjacent surface populations.

Variation in population densities can be explained by different factors affecting cave molly population dynamics; e.g., environmental heterogeneity may contribute to population differences. The highest H_2_S concentrations (>300 µM) are found in parts of chamber X, where most large sulfide sources are located, while concentrations in chamber V are lower (2–32 µM), as H_2_S is increasingly bound with oxygen with increasing distance from the sulfide sources (Tobler et al., 2006). However, ecotoxicological experiments repeatedly found small adults to have higher H_2_S-resistance than large-bodied adults, possibly reflecting senescence effects or size-specific thresholds regarding the rate of sulfide influx to the body to oxidation (Tobler et al., 2011b; Plath et al., 2013; Riesch et al., 2014a). Hence, we would expect fish in chamber X to actually be smaller than fish from chamber V if different H_2_S concentrations were the main driver of population differences.

Beside different H_2_S concentrations, the sites within the cave differ in the presence of light. Whereas chamber V receives some dim light through cracks in the ceiling, the inner parts of the cave are lightless. Photophobic behavior is a factor that has been proposed to promote the colonization of perpetually dark caves and the choice of microhabitat (Poulson, 1964; Barr Jr, 1968). While photophobic behavior has been reported in several cavefishes (Wilkens, 1988; Camassa, 2001; Wilkens, 2001; Timmermann & Plath, 2009), photophilic behavior was found in both surface and cave forms of *P. mexicana* (Parzefall et al., 2007). In theory, this photophilic behavior could lead to an accumulation of fish in chamber V (sites *5–7*) compared to chamber X (site *8*) if fish moved between cave chambers but were less likely to return to dark sites, but this line of argumentation is not compatible with the observation of small-scale genetic structure among different cave chambers (Plath et al., 2007).

The different light regimes may also affect trophic interactions since the deep and lightless parts of the cave depend solely on chemoautotrophic primary production, while organic matter can enter through cracks in chamber V, and then provide the basis for detritivore animal communities that constitute an additional food source in other cave systems (Hüppop, 2005). Nevertheless, more research is needed on the extent to which these few sky lights might indeed provide significant influx of additional nutrients, because stomach content analysis of cave mollies, for example, does not so far strongly support such a notion (Tobler et al., 2009).

Furthermore, cave chambers may differ in predation regimes. Inside the Cueva del Azufre, aquatic water bugs of the genus *Belostoma* prey upon cave mollies and *Belostoma* prefer large over small cave mollies as prey (Tobler, Schlupp & Plath, 2007; Tobler, Franssen & Plath, 2008; Plath et al., 2011). Mark-recapture analysis found individual densities of water bugs to be approximately one individual per m^2^ in chamber V (Tobler, Schlupp & Plath, 2007), and while empirical data are as yet lacking, observational evidence over several years of field work suggests that densities are much lower in the innermost chambers.

*Belostoma* predation, however, might explain microhabitat use of different size classes of cave mollies. *Belostoma* are typically found on rocks at the water’s edge (Tobler, Franssen & Plath, 2008), and so large cave mollies—being preferred by the water bugs (Plath et al., 2011)—could use deeper parts of the water column to avoid predation risk. The preference for large size-classes was confirmed for another belostomatid preying on mosquitofish (Schumann, Cavallaro & Hoback, 2012). On the other hand, small fish could avoid filial cannibalism, which is known from other poeciliids (Loekle, Madison & Christian, 1982; Nesbit & Meffe, 1993), by using shallow parts of the water column that exclude large mature fish.

One factor that most likely influences population dynamics is the annual ‘La Pesca’ ceremony. The ceremony leads to a strong temporary reduction of local fish densities in those cave chambers that are situated downstream of the barbasco release site (Tobler et al., 2011a). Our study was conducted approximately nine months after the last ceremony, but given rather long generation times in *P. mexicana* (roughly 3–6 months for males and 7–10 months for females from birth until reaching maturation under common-garden rearing conditions; Riesch et al., 2014b), we predicted to find lower (sub-)population densities and especially fewer large-bodied individuals downstream of the site in the Cueva del Azufre where barbasco is annually released. Instead, while fish densities were generally high in the cave, they were highest downstream of the barbasco release site. However, sample sites affected by the release of barbasco had population structures that were strongly shifted towards an overabundance of the smallest size classes (i.e., juveniles). These patterns were stable when repeated samplings from subsequent days were compared.

Migration within the Cueva del Azufre is unidirectional, out of the cave, and migration among different cave chambers occurs only to a small extent, which results in population genetic differentiation, as shown based on nuclear microsatellites (Plath et al., 2007), and is also reflected in morphological differences among fish from different cave chambers (Fontanier & Tobler, 2009). Hence, re-colonization of the affected sites from other parts of the cave (i.e., source–sink dynamics) is unlikely, and the observed recovery of the respective populations likely represents an autochthonous effect. After the temporal decline in population density following La Pesca, the surviving individuals benefit from reduced intraspecific resource competition. Detritus and green algae are the dominant food sources of surface-dwelling *P. mexicana* from non-sulfidic streams, while diets of conspecifics in the sulfidic surface and cave streams are dominated by chemoautotrophic (sulfur) bacteria and aquatic invertebrates (like larvae of the dipteran *Goeldichironomus fulvipilus* and small snails; (Roach, Tobler & Winemiller, 2011)). In particular, access to invertebrate prey could be favored not only by the absence of competing fish species, but especially by temporarily relaxed competition among the surviving adult *P. mexicana*. Generally, relaxed competition translates into higher growth rates, faster maturation, and increased adult fecundity (Stearns, 1976), which may lead to stage-specific biomass overcompensation, thereby compensating for the removal of individuals from the population (Werner & Gilliam, 1984; de Roos et al., 2007; Schröder, Persson & de Roos, 2009). This idea received support from empirical harvesting experiments that found the negative relationship between adult mortality and abundance/density to be reversed if mortality does not affect a certain portion of the population. Experimental studies on laboratory populations of the poeciliid fish *Heterandria formosa* showed that biomass of the juvenile size class increased in response to intermediate adult mortality rates (Schröder, Persson & de Roos, 2009). Another study showed that a pathogen outbreak in a wild perch population (*Perca fluviatilis*) was followed by a biomass overcompensation of the juvenile stage as a result of increased adult mortality. Age-specific adult fecundity and body mass of one- and two year old perch increased after the disease outbreak, suggesting that increased adult mortality released perch from competition and cannibalism, thereby increasing somatic and reproductive growth (Ohlberger et al., 2011). In the Cueva del Azufre, the stage-specific biomass overcompensation may lead to increasing population densities, based on temporarily increased adult fecundity that leads to high numbers of juvenile fish. This would result in cave molly populations regaining the high densities seen before La Pesca, again leading to increased competition. This is consistent with earlier observations of cave mollies showing reduced body condition (measured, e.g., as fat content) compared to fish from surface sites (Riesch, Plath & Schlupp, 2010; Riesch, Plath & Schlupp, 2011). Hence, human-induced selection and predation by *Belostoma* ought to have very similar effects on the populations exposed to them. We are inclined to argue, however, that the relative influence of *Belostoma* predation is considerably lower than the effects of the massive annual fish harvest. Previous reports of increased rotenone-resistance in fish from chamber V, but not chamber X (Tobler et al., 2011a), confirm that La Pesca undoubtedly has a strong selective influence on populations annually exposed to it.

In summary, we found remarkable fish densities of more than 200 individuals per m^2^ in some parts of the cave. While other selective forces certainly also need to be considered, we argue that the annual La Pesca has major effects on the population ecology and evolutionary trajectory of cave mollies. We are aware of potential caveats of this line of argument, as not all differences reported here may be due to the annual La Pesca ceremony. Nevertheless, from a conservational point of view, knowledge about whether and how human activities affect teleost populations is especially pertinent in the case of locally adapted populations that are endemic to a small area. Therefore, we recommend a repeated sampling before and after La Pesca in order to demonstrate the influence of the ritual. While human influences on highly endemic, locally adapted populations (or, in terms of conservation biology, evolutionary significant units; Moritz, 1994) generally are to be evaluated as highly problematic, management plans for cave mollies ought to consider the important role La Pesca plays in the religion of the local human population. Carried out in the traditional way, fish populations in downstream cave chambers can obviously recover after the ceremony. However, we wish to highlight the necessity to critically review that those practices do not affect deeper parts of the cave and that no commercially available, more efficient fish toxins will be employed in the future.

## Supplemental Information

10.7717/peerj.490/supp-1 Supplemental Information 1 Raw data

## Additional Information and Declarations

### Field Study Permissions

The following information was supplied relating to field study approvals (i.e., approving body and any reference numbers): Current research was authorized by CONAPESCA-DGOPA.09004.041111.3088 and the Tacotalpa, Tabasco municipality.
